# Association of adipokines with major adverse cardiovascular events following acute myocardial infarction: a systematic review and meta-analysis

**DOI:** 10.1186/s12872-026-06120-0

**Published:** 2026-06-12

**Authors:** Auda Nadira, Sunita Sunita, Bima Diokta Alparisi, Amanda Cheryl Fayza, Nur Syifa Rahmatika, Putri Karisa, Nova Sylviana, Mas Rizky A.A Syamsunarno

**Affiliations:** 1 Master Study Program of Biomedical Sciences, Faculty of Medicine, Universitas Padjadjaran, Bandung, West Java Indonesia; 2 Department of Biomedical Sciences, Faculty of Medicine Universitas Padjadjaran, Bandung, West Java Indonesia; 3 Cardiometabolic Working Group Study, Faculty of Medicine, Universitas Padjadjaran, Bandung, West Java Indonesia; 4 Doctoral Program, Faculty of Medicine, Universitas Padjadjaran, Bandung, West Java Indonesia; 5 Bachelor Study Program, Faculty of Medicine, Universitas Padjadjaran, Bandung, West Java Indonesia; 6 Department of Medical Laboratory Technology, Poltekkes Kemenkes Bengkulu, Bengkulu, Indonesia; 7 Clinical Professional Program, RSUD Arifin Achmad, Faculty of Medicine, Universitas Riau, Pekanbaru, Riau, Indonesia

**Keywords:** Acute myocardial infarction, Adipokine, Adiponectin, Visfatin, Major adverse cardiovascular events

## Abstract

**Background:**

Major adverse cardiovascular events (MACEs) remain a leading cause of poor prognosis following acute myocardial infarction (AMI). Emerging evidence suggests that adipose tissue-derived adipokines may provide additional information on cardiometabolic risk beyond conventional anthropometric measures. This systematic review and meta-analysis aimed to evaluate the association between circulating adipokines, specifically adiponectin and visfatin, and the occurrence of MACE among patients with AMI.

**Methods:**

A systematic search of PubMed/MEDLINE, Scopus, and Cochrane Library was performed from inception to identify cohort studies reporting adiponectin or visfatin levels in adult AMI patients with and without MACE during follow-up. Pooled mean differences (MD) with 95% confidence intervals (CI) were calculated.

**Results:**

Five cohort studies (*n* = 707 AMI patients) conducted across China, Taiwan, Japan, and Poland, with follow-up durations ranging from 2 to 43 months, were included. Adiponectin levels were lower in MACE patients (MD=-2.85 [95% CI -5.42 to -0.27, *p* = 0.03]; I^2^ = 94%), while visfatin levels were significantly higher in the MACE group (MD = 2.99 [95% CI 1.51 to 4.47, *p* < 0.0001]; I^2=^0%).

**Conclusion:**

Lower adiponectin and higher visfatin levels are associated with MACE occurrences in AMI patients, whereas BMI did not demonstrate significant discriminatory value.

**Supplementary Information:**

The online version contains supplementary material available at 10.1186/s12872-026-06120-0.

## Introduction

Improving cardiovascular health and reducing premature mortality remain major global health priorities [1]. Acute myocardial infarction (AMI) contributes substantially to this burden, with cardiovascular diseases accounting for approximately 19.8 million deaths globally in 2022, most of which were attributable to myocardial infarction and stroke [2]. Despite advances in acute management and early revascularization, major adverse cardiovascular events (MACE) following AMI remain common and remain associated with substantial long-term morbidity and mortality [3].

Several studies have described an obesity paradox in acute coronary syndromes (ACS), where patients with higher body mass index (BMI) appear to have better survival rates than those with normal weight [4]. This paradox has raised interest in the qualitative characteristics of adipose tissue rather than fat quantity, particularly its endocrine activity through the secretion of adipokines.

Adipose tissue is now recognized as an active endocrine organ that releases a wide range of bioactive mediators, known as adipokines, which modulate vascular inflammation, endothelial function, and myocardial remodeling. Among these, adiponectin and visfatin have attracted particular attention due to their prominent, partially opposing roles in cardiovascular pathology [5]. Adiponectin is generally considered an antiinflammatory and vasculoprotective adipokine that improves endothelial function, attenuates plaque formation, and inhibits cardiomyocyte apoptosis [6]. In contrast, visfatin exerts predominantly pro inflammatory and pro atherogenic effects, contributing to endothelial dysfunction, plaque instability, and adverse ventricular remodeling [7, 8].

Despite growing interest in adipokines following AMI, current clinical evidence remains limited and inconsistent. Previous studies evaluating adiponectin and visfatin in post-AMI patients have reported heterogeneous associations with MACE, potentially related to differences in study populations, timing of biomarker measurements, follow-up duration, and MACE definitions [9–13]. Furthermore, the potential role of circulating adiponectin and visfatin in the chronic phase after AMI remained unclear. Addressing these gaps was crucial, as understanding the predictive value of adipokines could guide personalized follow-up strategies and therapeutic interventions beyond standard clinical assessment. Therefore, this systematic review and meta-analysis aimed to evaluate the association between circulating adiponectin and visfatin levels and MACE incidence in AMI patients. This approach not only synthesized current evidence, but also sought to highlight inconsistencies and gaps in the literature, providing a foundation for future research and clinical applications.

## Methods

### Study design and registration

This systematic review and meta-analysis followed the Preferred Reporting Items for Systematic Reviews and Meta-Analysis (PRISMA) (Table S1 and S2) guidelines and was registered in the International Prospective Register of Systematic Reviews (PROSPERO; registration number CRD420251271611) [14].

### Search strategy and study selection

Relevant studies were searched in Medline (via PubMed), Scopus, and the Cochrane Library. All peer-reviewed studies from inception to the latest available publications were included, without restrictions on language or geographical region. A combination of Medical Subject Headings (MeSH) and free-text terms was used as keywords in the search strategy, focusing on “adipokine”, “adiponectin”, “visfatin”, “major adverse cardiovascular events”, “acute myocardial infarction”, and their synonyms. The search strategy was adapted for each database according to its indexing system and search syntax. For the Cochrane Library, an all-fields search approach was applied to maximize retrieval sensitivity. Further details of the search strategy are provided separately (Table S3).

### Research question

The research question was: “Among patients with acute myocardial infarction (AMI) (P), are circulating adipokine levels, particularly adiponectin and visfatin (E), associated with the occurrence of major adverse cardiovascular event (MACE) during follow-up (O) compared with patients who did not develop MACE (C)?”.

### Inclusion criteria

The inclusion criteria were structured according to the PECO (Population, Exposure, Comparison, Outcome) framework. The population (P) consisted of adult patients diagnosed with acute myocardial infarction (AMI), including STEMI and NSTEMI, who were followed longitudinally for the occurrence of major adverse cardiovascular event (MACE). The exposure (E) was defined as circulating adipokine levels, particularly adiponectin and visfatin. Circulating adipokine levels were assessed quantitatively adiponectin and visfatin concentrations as reported in each study. The comparison (C) consisted of AMI patients who did not develop MACE during follow-up. The outcome (O) was defined as the occurrence of MACE during follow-up, including cardiovascular death, recurrent myocardial infarction, coronary revascularization, heart failure, stroke, or hospitalization for cardiovascular causes according to each study definition. Studies were required to include a comparison between AMI patients who developed MACE and those who did not during follow-up. Eligible study designs included retrospective or prospective observational cohort studies reporting adiponectin or visfatin levels in relation to MACE occurrence.

Because the included studies compared adipokine levels between patients with and without MACE during follow-up, the findings should be interpreted as exploratory associations rather than causal or temporal relationships. Therefore, reverse causality cannot be excluded.

### Exclusion criteria

Studies were excluded if they were cross-sectional, case reports, small case series (fewer than 5 patients), editorials, commentaries, reviews, meta-analyses, conference proceedings, interventional studies, case-control studies, grey literature, or unpublished studies assessing adipokines and MACE in AMI patients.

### Data extraction and quality assessment

The following data were extracted from each included study: first author, publication year, country, study design, type of AMI, sample size, patient characteristics, follow-up duration, MACE definition, timing of blood sampling, adipokine measurements especially adiponectin and visfatin, body mass index (BMI), and reported outcome data for patients with and without MACE. All articles identified through the search strategy were uploaded to Rayyan (www.rayyan.ai), an online platform for systematic reviews. After removing duplicates, all titles and abstracts were screened for eligibility, and the full texts of relevant studies were independently assessed by two authors (AN, NRS); reasons for study exclusion were recorded. Any disagreements between the reviewers were resolved by consensus, and if disagreement persisted, a third author (MRAAS) was consulted. The risk of bias of the included studies was assessed independently by two authors (AN and ACF) using the Risk Of Bias In Non-randomized Studies of Interventions (ROBINS-I) tool [15]. The other authors (BDA, SS, PK, NS, and MRAAS) were responsible for supervising the overall content.

### Statistical analysis

Statistical analysis was performed using RevMan software (version 5.3; RevMan International, Inc., New York, NY, USA). Continuous outcomes were pooled using mean difference (MD) with 95% confidence interval (CI) based on the inverse-variance estimator. Statistical heterogeneity was assessed using Cochran’s Q test and quantified using the inconsistency index (I^2^). A p-value of < 0.10 in Cochran’s Q test was considered indicative of significant heterogeneity. A random-effects model was applied when substantial heterogeneity was detected (I^2^ > 50%); otherwise, a fixed-effect model was used. Hartung-Knapp adjustment was not applied because of limited number of included studies and software constraints in RevMan 5.3.

For studies reporting continuous variables as median and interquartile range (IQR), data were converted to mean ± standard deviation (SD) using established methods. Publication bias assessment using forest plots was not performed because the number of included studies was fewer than ten, which limits the reliability of asymmetry testing [16–19].

## Results

### Study selection

**Fig. 1:**
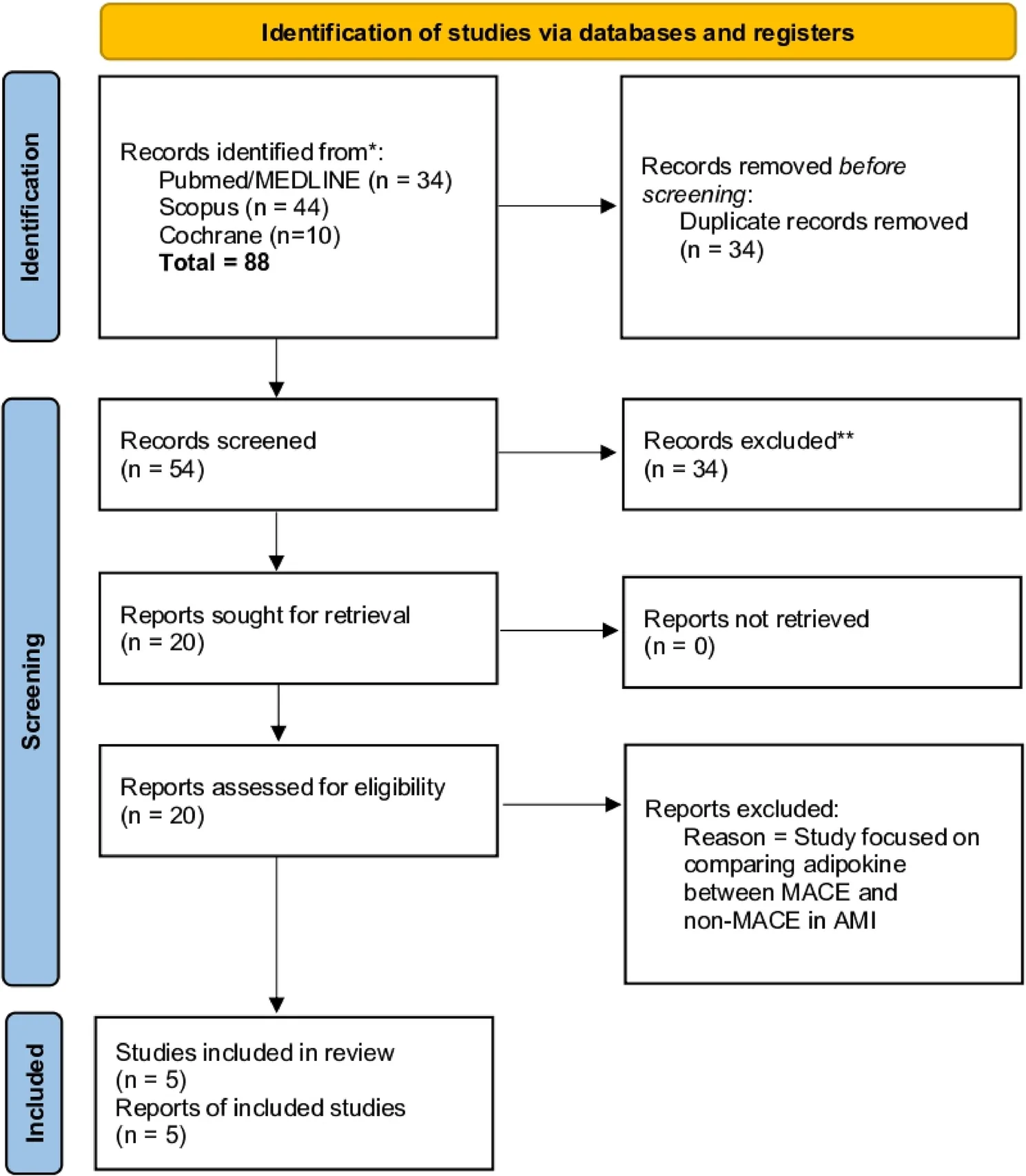
PRISMA flow chart. *In this flowchart, records are tracked only through databases (no registries). **Based on the study’s exclusion criteria, articles that contained only a review or an abstract of grey literature were not included

A total of 88 studies were identified from PubMed (*n* = 34), Scopus (*n* = 44), and Cochrane Library (*n* = 10). After duplicate removal, 34 studies were excluded, leaving only 54 articles for screening. Furthermore, 34 studies were excluded based on title and abstract, leaving 20 studies eligible for full-text review. Of the 20 studies assessed for full-text eligibility, 15 were excluded for failing to meet the inclusion criteria because they did not report adipokine levels between MACE and non-MACE. Finally, 5 studies were included in this systematic review as shown in the PRISMA flowchart (Fig. 1).

### Characteristics of included studies

A total of 707 AMI patients were included (192 patients with MACE and 515 patients without MACE). All five studies employed prospective cohort designs and enrolled patients with STEMI and/or NSTEMI. Details of the included studies are presented in Table 1, while baseline clinical characteristics of the MACE and non-MACE groups, including age, sex distribution, cardiovascular risk factors, serum creatinine levels, and medication use, are summarized in Table 2. The follow-up duration ranged from 2 to 43 months. Most studies were conducted in Asian populations, including China, Taiwan, and Japan, with only one study originating from Europe (Poland), which may limit the generalizability of the findings. Definitions of MACE varied across studies and included different combinations of cardiovascular death, myocardial infarction, coronary revascularization, heart failure, unstable angina, and stroke. In addition, the timing of adipokine measurement differed substantially across studies, ranging from the acute phase of AMI to the chronic phase at 18 months post-AMI, which may have contributed to heterogeneity in pooled estimates. The five included studies assessed adiponectin (*n* = 3) and visfatin (*n* = 2). Adiponectin (µg/mL) was measured during the acute phase on day 1 after admission in the fasting state (MACE 3.97 ± 0.88 vs. non-MACE 9.93 ± 6.39) and acute phase before PCI (MACE 9.0 ± 0.6 vs. non-MACE 7.9 ± 0.7), while during the chronic phase at 18 months post-AMI levels were 4.01 ± 2.40 (MACE) vs. 5.78 ± 4.64 (non-MACE). Visfatin (ng/mL) was measured in two acute-phase studies: on admission (MACE 20.07 ± 15.21 vs. non-MACE 15.33 ± 12.52) and 8–12 h after admission in the fasting state prior to coronary angiography (MACE 10.78 ± 2.53 vs. non-MACE 7.67 ± 1.81).

**Table 1:** Study characteristics

Studies	Country	Study Design	AMI type	Total sample (*n*,%)		Follow-up* (months)	MACE Definition	Timing of blood sampling	Adipokine
				MACE	Non-MACE				
Huang (2010)	Taiwan	Prospective Cohort	Acute MI (STEMI and NSTEMI)	30 (29.4%)	72 (70.6%)	43 ± 12	CV death, MI, CR, Stroke, Unstable angina	Chronic phase (At 18 months post-AMI)	Adiponectin
Piestrzeniewicz (2008)	Poland	Prospective Cohort	STEMI	9 (11.7%)	68 (88.3%)	12	CV death, MI, Unstable angina, HF	Acute phase (Fasting, day 1 after admission)	Adiponectin
Shioji (2007)	Japan	Prospective Cohort	Acute MI (STEMI and NSTEMI)	78 (42.4%)	106 (57.6%)	27.3	All-cause death, MI, CR, HF, Stroke	Acute phase (Before PCI)	Adiponectin
Hung (2015)	Taiwan	Prospective Cohort	STEMI	57 (30.8%)	128 (69.2%)	Median 8 (IQR: 2–20)	Death, MI, CR, HF	Acute phase (On admission)	Visfatin
Zheng (2020)	China	Prospective Cohort	Acute MI (STEMI and NSTEMI)	18 (11.3%)	141 (88.7%)	Mean 19.3 (16–24)	CV death, MI, CR, HF	Acute phase (Fasting, 8–12 h before coronary angiography)	Visfatin

Follow-up duration (months) was reported as mean, mean ± SD, or median/mean (IQR/range)

*Abbreviations*: *AMI* acute myocardial infarction, *BMI* body mass index, *CR* coronary revascularization, *CV* cardiovascular, *HF* heart failure, *IQR* interquartile range, *MACE* major adverse cardiovascular events, *MI* myocardial infarction, *NSTEMI* non–ST-segment elevation myocardial infarction, *PCI* percutaneous coronary intervention, *SD* standard deviation, *STEMI* ST-segment elevation myocardial infarction, *h* hours, *n* number of participants

**Table 2:** Clinical characteristics

	Huang (2010)		Piestrzeniewicz (2008)		Shioji (2007)		Hung (2015)		Zheng (2020)	
	MACE (*n* = 30)	Non-MACE (*n* = 72)	MACE (*n* = 9)	Non-MACE (*n* = 68)	MACE (*n* = 78)	Non-MACE (*n* = 106)	MACE (*n* = 57)	Non-MACE (*n* = 128)	MACE (*n* = 18)	Non-MACE (*n* = 141)
Age (years)	63 ± 11	62 ± 11	57 ± 6.69	53.99 ± 6.77	66.9 ± 1.3	65.6 ± 1.1	68.5 ± 12.6	61.8 ± 13.2	68.89 ± 8.99	62.23 ± 10.58
Male sex (n, %)										
Male	24 (80%)	63 (88%)	NR	NR	60 (76.9%)	67 (63.2%)	42 (73.7%)	101 (78.9%)	13 (72.22%)	122 (86.52%)
Female	6 (20%)	9 (12%)	NR	NR	18 (23.1%)	39 (36.8%)	15 (26.3%)	27 (21.1%)	5 (27.78%)	19 (13.48%)
BMI (kg/m^2^)	26 ± 3	26 ± 4	28.25 ± 5.16	27.96 ± 4.51	23.9 ± 0.4	24.0 ± 0.4	23.9 ± 3.6	24.8 ± 3.9	24.35 ± 2.55	33.85 ± 2.98
Hypertension (n, %)	18 (60%)	25 (49%)	7 (78%)	34 (50%)	41.6%*	44.2%*	36 (63.2%)	75 (58.6%)	13 (72.22%)	70 (49.64%)
Diabetes melitus (n, %)	15 (50%)	20 (28%)	7 (78%)	10 (15%)	47.4%*	26.4%*	30 (52.6%)	40 (31.3%)	9 (50%)	32 (22.69%)
Dyslipidemia (n, %)	10 (33%)	27 (38%)	8 (89%)	54 (79%)	NR	NR	41 (71.9%)	95 (74.2%)	3 (16.67%)	44 (31.2%)
Smoking (n, %)	15 (50%)	41 (57%)	8 (89%)	43 (63%)	49.3%*	39.6%*	24 (55.8%)	34 (45.3%)	8 (44.44%)	78 (52.48%)
Creatinine	NR	NR	0.96 ± 0.22 mg/dL	0.95 ± 0.16 mg/dL	NR	NR	137.3 ± 91.5 umol/L	106.8 ± 53.4 umol/L	101.67 ± 46.63 umol/L	79.88 ± 22.59 umol/L
Medication										
Antiplatelet agent (n, %)	28 (93%)	71 (99%)	9 (100%)	68 (100%)	NR	NR	NR	NR	NR	NR
β-blocker (n, %)	19 (63%)	50 (69%)	8 (89%)	66 (97%)	41%*	40.6%*	NR	NR	NR	NR
Statin (n, %)	12 (40%)	42 (58%)	9 (100%)	68 (100%)	50%*	64.2%*	35 (61.4%)	72 (56.3%)	NR	NR
ACEi/ARB (n, %)	24 (80%)	56 (78%)	7 (78%)	39 (57%)	74.4%*	67.9%*	NR	NR	NR	NR
Adiponectin (µg/mL)	4.01 ± 2.40	5.78 ± 4.64	3.97 ± 0.88	9.93 ± 6.39	7.9 ± 0.7	9.0 ± 0.6	NR	NR	NR	NR
Visfatin (ng/mL)	NR	NR	NR	NR	NR	NR	20.07 ± 15.21	15.33 ± 12.52	10.45 ± 3.33	7.67 ± 1.81

Values are the mean ± SD or number (%); *only percentages were reported in the original study

*Abbreviations*: *NR* not reported, *BMI* body mass index, *ACEi* angiotensin-converting enzyme inhibitor, *ARB* angiotensin II receptor blocker, *µg/mL* microgram per milliliter, *ng/mL* nanogram per milliliter, *n* number of participants

### Association of adipokine and incidence of MACE in AMI

#### Adiponectin

**Fig. 2:**
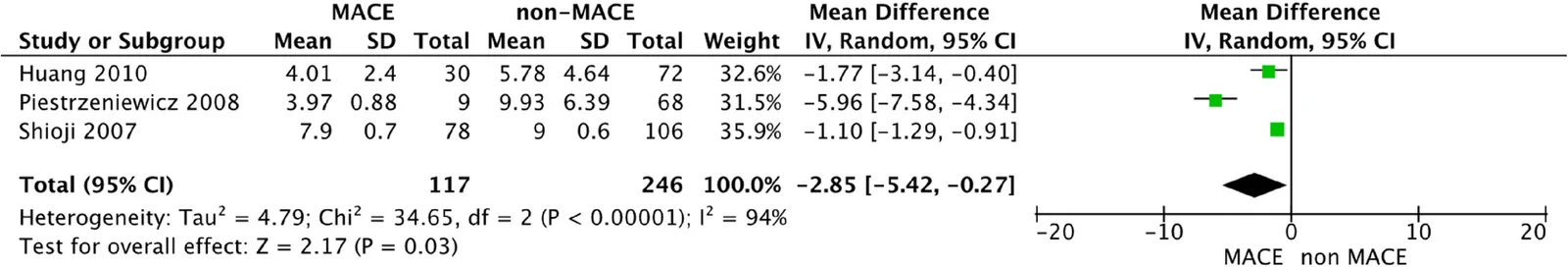
Forest plot of adiponectin levels in AMI patients with and without MACE

Adiponectin was analyzed in three studies. As shown in Fig. 2, adiponectin levels were significantly lower in MACE patients than in non-MACE (MD = -2.85 [95% CI -5.42 to -0.27, *p* = 0.03]). However, heterogeneity was also markedly high (I² = 94%, p_heterogeneity_ < 0.00001), and the findings should therefore be interpreted cautiously because of substantial heterogeneity and the limited number of included studies.

#### Visfatin

**Fig. 3:**
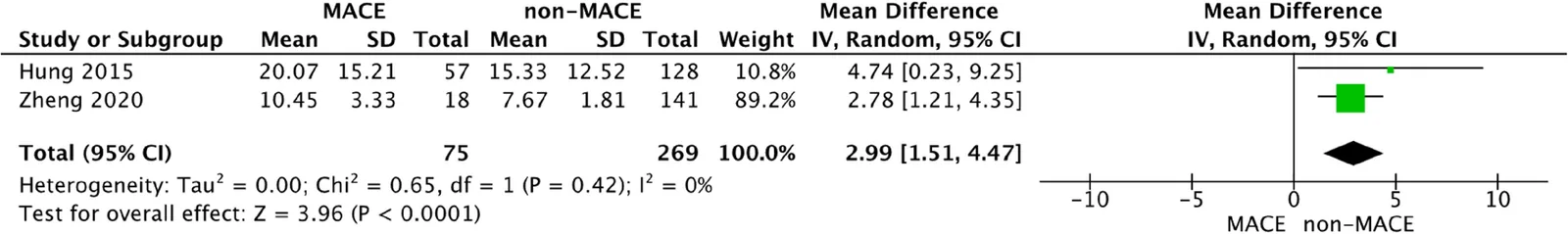
Forest plot of visfatin levels in AMI patients with MACE and without MACE

Visfatin was analyzed in two studies. As shown in Fig. 3, visfatin levels were significantly higher in AMI patients with MACE than in those without MACE (MD = 2.99 [95% CI 1.51 to 4.47, *p* < 0.0001]). We found no evidence of significant heterogeneity (I² = 0%, p_heterogeneity_ =0.42). However, the findings should be interpreted cautiously because only two studies were included in the pooled analysis.

### Cardiometabolic risk characteristics

**Fig. 4:**
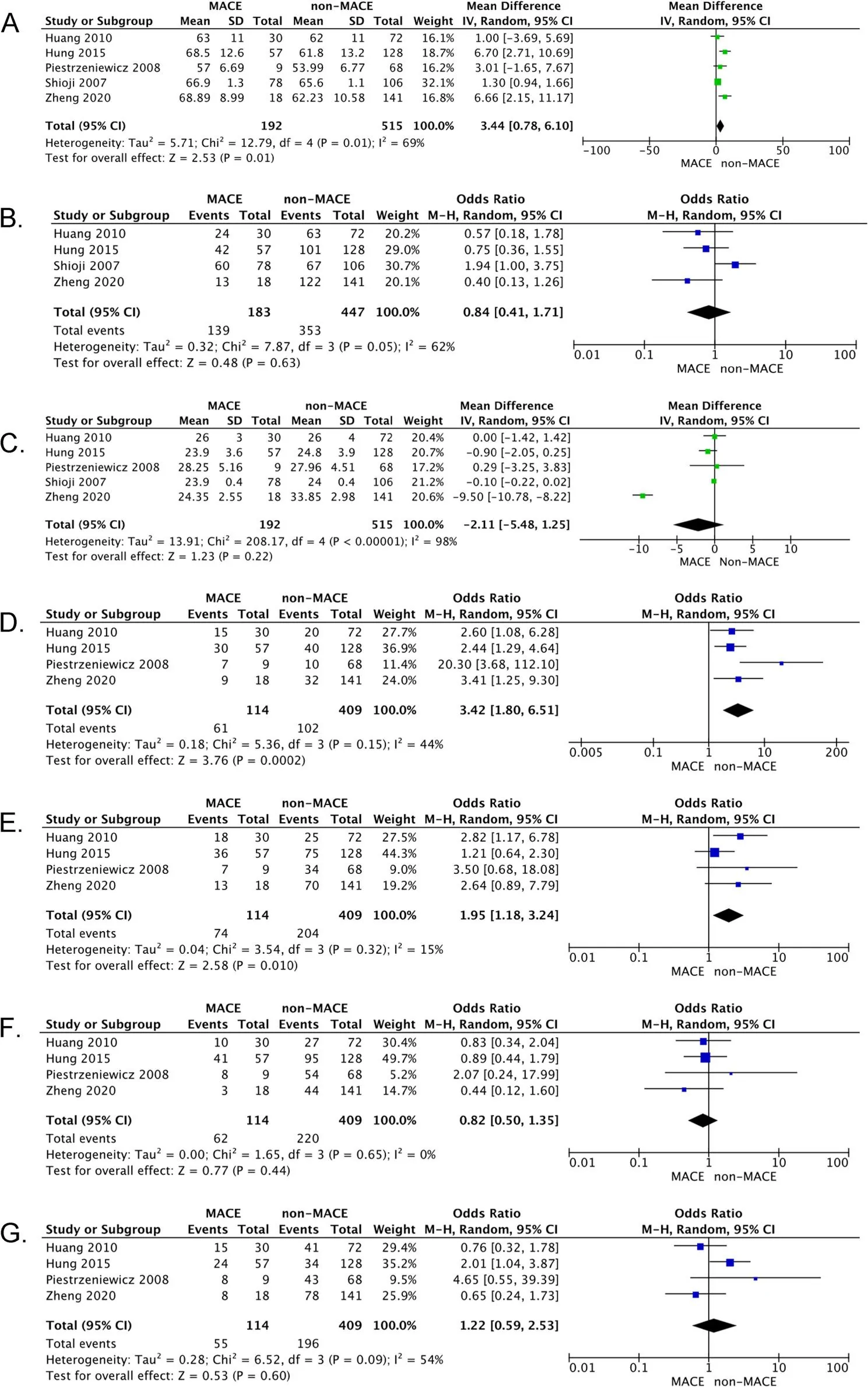
Forest plots of pooled baseline characteristics comparing AMI patients with and without MACE: **A** Age; **B** Male Sex; **C** BMI; **D** Diabetes melitus; **E** Hypertension; **F** Dyslipidemia; **G** Smoking

The pooled analyses demonstrated significant differences in age, diabetes mellitus, and hypertension between AMI patients with and without MACE. The results of the five studies included in the age analysis are shown in Fig. 4A. Patients with MACE were significantly older than those without MACE (MD = 3.44 [95% CI 0.78 to 6.10, *p* = 0.01]). However, high heterogeneity was observed (I^2^ = 69%, p_heterogeneity_ = 0.01).

The results of the four studies included in the male sex analysis are shown in Fig. 4B. No significant difference in male sex distribution was observed between the two groups (OR = 0.84 [95% CI 0.41 to 1.71, *p* = 0.63]). High heterogeneity was observed (I^2^ = 62%, p_heterogeneity_ = 0.05).

The results of the five studies included in the body mass index (BMI) analysis are shown in Fig. 4C. No significant difference in BMI was observed between the two groups (MD = -2.11 [95% CI -5.48 to 1.25, *p* = 0.22]). However, high heterogeneity was observed (I^2^ = 98%, p_heterogeneity_ < 0.00001).

The results of the four studies included in the diabetes mellitus analysis are shown in Fig. 4D. Diabetes mellitus was significantly more prevalent among AMI patients with MACE compared with those without MACE (OR = 3.42 [95% CI 1.80 to 6.51, *p* = 0.0002]). Low heterogeneity was observed (I^2^ = 44%, p_heterogeneity_ = 0.14).

The results of the four studies included in the hypertension analysis are shown in Fig. 4E. Hypertension was significantly more prevalent among AMI patients with MACE compared with those without MACE (OR = 1.95 [95% CI 1.18 to 3.24, *p* = 0.01]). Low heterogeneity was observed (I^2^ = 15%, p_heterogeneity_ = 0.32).

The results of the four studies included in the dyslipidemia analysis are shown in Fig. 4F. No significant difference in dyslipidemia prevalence was observed between the two groups (OR = 0.82 [95% CI 0.50 to 1.35, *p* = 0.44]). Low heterogeneity was observed (I^2^ = 0%, p_heterogeneity_ = 0.65).

The results of the four studies included in the smoking analysis are shown in Fig. 4G. No significant difference in smoking status was observed between the two groups (OR = 1.22 [95% CI 0.59 to 2.53, *p* = 0.60]). High heterogeneity was observed (I^2^ = 54%, p_heterogeneity_ = 0.09).

Overall, these pooled cardiometabolic findings should be interpreted cautiously because several analyses were based on a limited number of studies and demonstrated substantial heterogeneity.

### Quality of the studies

**Fig. 5:**
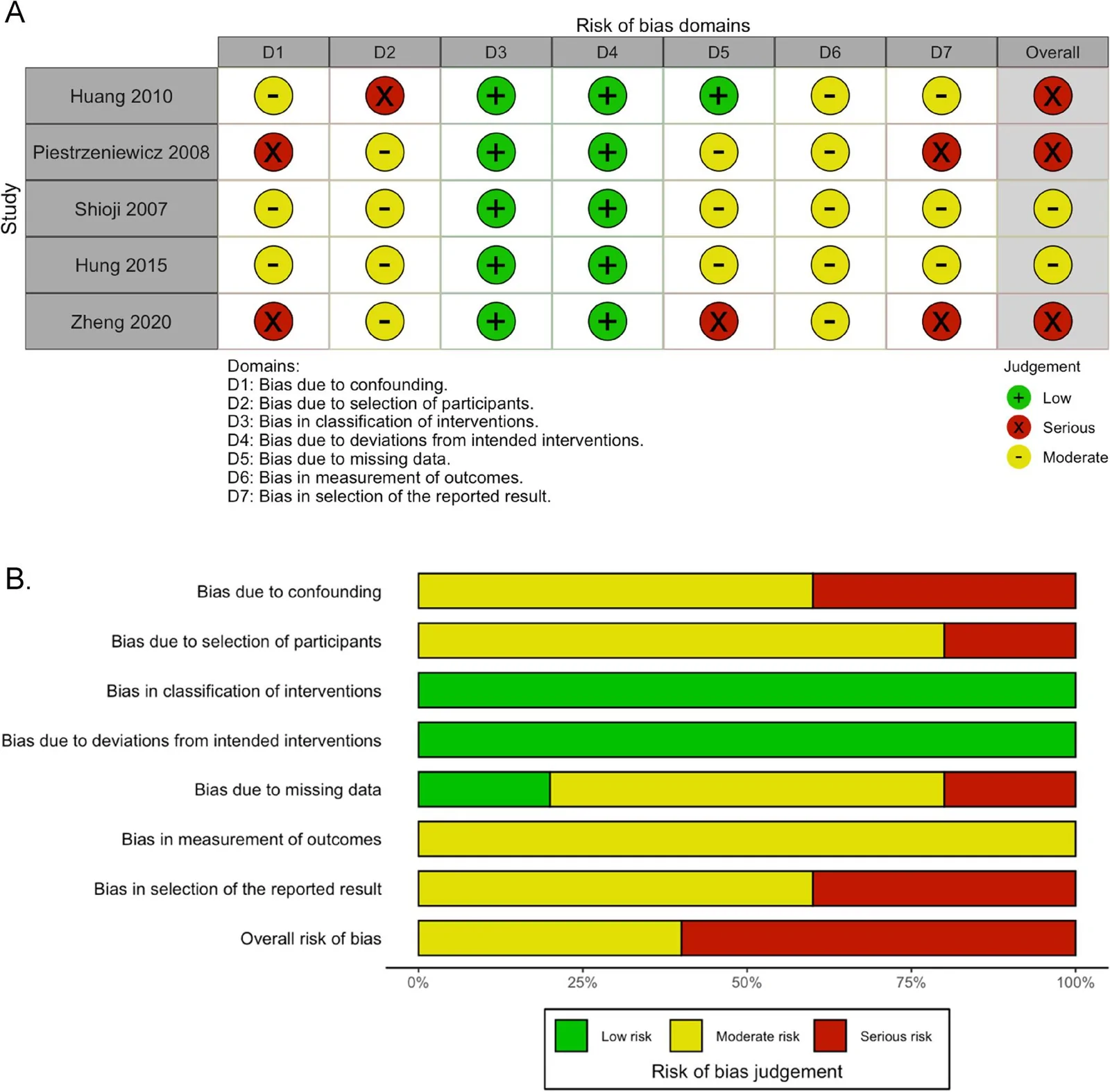
ROBINS-I risk of bias assessment, **A** RoB Traffic Light; **B** RoB Summary

The quality of the included studies was assessed using the ROBINS-I tool, and the detailed results are presented in Fig. 5 and Table S4 [15]. Overall, the risk of bias across studies ranged from moderate to serious. Two studies (Huang 2010 and Zheng 2020) [9, 12] were judged as having an overall serious risk of bias, while the remaining studies demonstrated moderate overall risk of bias.

Most studies demonstrated low risk of bias in the classification of interventions (D3) and deviations from intended interventions (D4). However, moderate to serious concerns were identified in several other domains, particularly bias due to confounding (D1), selection of participants (D2), missing data (D5), and selection of the reported result (D7). Serious risk of bias due to confounding was observed in Piestrzeniewicz 2008 [13] and Zheng 2020 [9], likely related to limited adjustment for baseline cardiovascular risk factors and comorbidities.

Moderate risk of bias in outcome measurement (D6) was consistently observed across all studies, reflecting potential variability in adipokine measurement methods and timing of blood sampling. In addition, serious concerns regarding selective reporting (D7) were identified in Piestrzeniewicz 2008 [13] and Zheng 2020 [9]. These findings suggest that methodological heterogeneity and residual confounding may have influenced the pooled estimates and should be considered when interpreting the results of this meta-analysis.

### Grading of Recommendations Assessment, Development, and Evaluation (GRADE) assessment

Certainty of evidence was assessed using the GRADE approach [20]. The evaluation of adipokines and cardiometabolic risk characteristics revealed overall low to very low certainty of evidence, reflecting important limitations within the currently available evidence base. All included outcomes were derived from observational studies, resulting in a serious risk of bias due to potential confounding factors and selection bias.

Moderate to high heterogeneity was observed across several pooled analyses, particularly for adiponectin, BMI, age, male sex, and smoking status, and these outcomes were subsequently downgraded for inconsistency. Adiponectin and BMI demonstrated very serious inconsistency because of markedly high heterogeneity across studies. Variability in MACE definitions, timing of adipokine measurements, study populations, baseline cardiometabolic characteristics, and methodological differences may have contributed to the observed heterogeneity and limited the generalizability of pooled estimates.

Imprecision was also identified in adiponectin and visfatin analyses because of the limited number of included studies and relatively small sample sizes. However, no serious indirectness was identified because the included studies directly evaluated AMI patients with and without MACE and assessed clinically relevant adipokine and cardiometabolic outcomes. Formal assessment of publication bias was limited because fewer than 10 studies were included in each pooled analysis; therefore, publication bias could not be adequately evaluated.

Overall, adiponectin, visfatin, and BMI were rated as very low-certainty evidence, whereas age, male sex, diabetes mellitus, hypertension, dyslipidemia, and smoking were rated as low-certainty evidence. These findings suggest that the currently available evidence should be interpreted cautiously and considered exploratory rather than definitive. Further large-scale, longitudinal, and standardized studies are required to validate these findings and clarify the role of adipokines and cardiometabolic risk factors in MACE among AMI patients (Table S5).

### Inconsistency and heterogeneity

The forest plots (Figs. 2 and 4A-C, and 4G) demonstrated heterogeneous patterns and variable effect estimates across pooled adipokine and cardiometabolic analyses in AMI patients with and without MACE. The moderate to high heterogeneity observed in several analyses suggests substantial variability between included studies. Such heterogeneity may reflect differences in study populations, baseline cardiometabolic characteristics, MACE definitions, timing of adipokine measurements, and methodological variations across studies.

Adiponectin demonstrated markedly high heterogeneity (I² = 94%), although most included studies consistently reported lower adiponectin levels in AMI patients with MACE compared with those without MACE. Similarly, BMI analysis showed very high heterogeneity (I² = 98%), despite pooled estimates generally demonstrating lower BMI in MACE patients. Variability in metabolic status, obesity prevalence, and study populations may have contributed to these inconsistent effect sizes.

Moderate heterogeneity was also identified in age, male sex, and smoking analyses. Age was consistently higher in AMI patients with MACE, although the magnitude of pooled differences varied across studies. Male sex showed inconsistent effect estimates, potentially related to variable sex distributions and incomplete reporting in some cohorts. Smoking analysis also demonstrated moderate heterogeneity, likely because definitions of smoking status were not consistently reported across studies and smoking duration or intensity were not clearly described.

In contrast, lower heterogeneity was observed in diabetes mellitus, hypertension, dyslipidemia, and visfatin analyses, suggesting relatively more consistent pooled findings across studies. However, these findings should still be interpreted cautiously because the number of included studies was limited, particularly for visfatin analysis, which included only two studies. Therefore, even statistically significant pooled estimates with low heterogeneity may have limited robustness and generalizability.

Overall, statistical significance does not necessarily indicate clinical relevance, particularly in the presence of substantial heterogeneity, limited sample sizes, and variable effect estimates. Standardized definitions of MACE, harmonized adipokine measurement protocols, and larger longitudinal studies will be important to improve the generalizability and clinical applicability of adipokine and cardiometabolic risk assessments in AMI patients (Table S6).

## Discussion

### Principal findings

This systematic review and meta-analysis synthesized the available prospective cohort evidence on the association between circulating adipokines and major adverse cardiovascular events (MACE) following acute myocardial infarction (AMI). The principal findings demonstrated that AMI patients who developed MACE exhibited significantly lower adiponectin levels and significantly higher visfatin levels compared with those without MACE. In addition, pooled cardiometabolic analyses showed that patients with MACE were older and had a higher prevalence of diabetes mellitus and hypertension. In contrast, no significant differences were observed for male sex, body mass index (BMI), dyslipidemia, or smoking status. The findings of this study indicate that circulating adipokine profiles may be associated with adverse post-AMI outcomes and may reflect cardiometabolic and inflammatory vulnerability not captured by conventional anthropometric measures. However, it is important to note that these results should be interpreted strictly as associations rather than evidence of causality. This is due to the observational design of the included studies and the limited number of available cohorts.

The observed association between lower adiponectin levels and MACE is biologically plausible considering the protective cardiovascular properties of adiponectin. Adiponectin is an anti-inflammatory and insulin-sensitizing adipokine that activates AMP-activated protein kinase (AMPK), endothelial nitric oxide synthase (eNOS), and peroxisome proliferator-activated receptor-alpha (PPAR-α), thereby promoting fatty acid oxidation, endothelial homeostasis, and suppression of oxidative stress and inflammatory signaling [21]. Reduced adiponectin concentrations have been associated with endothelial dysfunction, insulin resistance, vascular inflammation, impaired myocardial metabolism, and accelerated atherosclerosis. In the context of AMI, lower adiponectin levels in AMI patients with MACE may be associated with impaired cardioprotective and anti-inflammatory responses during myocardial ischemia and post-infarction remodeling, potentially contributing to MACE [22]. The substantial heterogeneity observed for adiponectin further indicates that the pooled estimate may have been influenced by differences in patient characteristics, AMI subtype, comorbidity burden, treatment strategies, follow-up duration, and laboratory measurement protocols.

Similarly, the finding of elevated visfatin levels among AMI patients with MACE is supported by its pro-inflammatory and pro-atherogenic properties. Visfatin, also known as extracellular nicotinamide phosphoribosyltransferase (eNAMPT), is secreted predominantly by visceral adipose tissue and activated inflammatory cells. Experimental studies suggest that visfatin promotes endothelial dysfunction, oxidative stress, vascular inflammation, matrix degradation, and plaque instability through activation of NF-κB, MAPK, and inflammatory cytokine pathways [23, 24]. Elevated visfatin concentrations have also been associated with myocardial injury severity, coronary plaque vulnerability, and adverse cardiovascular prognosis. The low statistical heterogeneity observed in the pooled visfatin analysis suggests relatively consistent findings across included studies. Nevertheless, interpretation remains limited because only two studies were available for pooled analysis.

Interestingly, despite significant differences in adipokine profiles, BMI did not differ significantly between AMI patients with and without MACE. This finding highlights the potential limitation of BMI as a surrogate marker of cardiometabolic risk in post-AMI populations. BMI does not adequately distinguish visceral adiposity, ectopic fat accumulation, body composition, or adipose tissue inflammatory activity, all of which may more directly influence adipokine secretion and cardiovascular risk. The absence of a significant BMI difference may suggest that adipose tissue dysfunction and inflammatory adipokine dysregulation are more relevant to post-AMI prognosis than conventional anthropometric measures [25]. In contrast, diabetes mellitus and hypertension were significantly more prevalent among patients with MACE, suggesting that underlying metabolic and vascular dysfunction may contribute to both altered adipokine profiles and adverse cardiovascular outcomes.

**Fig. 6:**
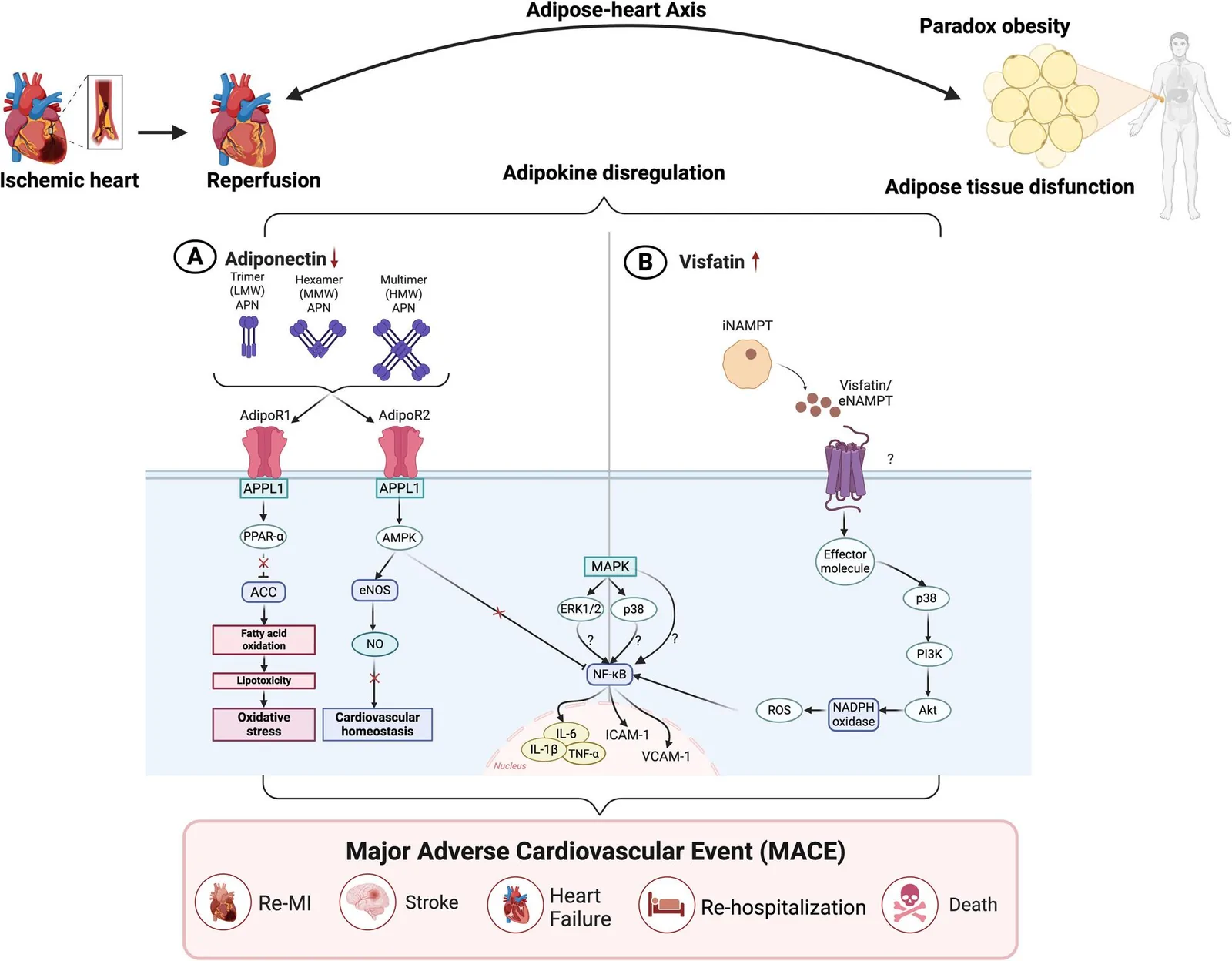
Proposed mechanism linking adipokine dysregulation and major adverse cardiovascular events following acute myocardial infarction

Our findings highlight the gap between the mechanistic understanding of adipokine signaling and clinical prognostic evidence. Although experimental studies support bidirectional communication between adipose tissue and the heart, forming the basis of the proposed adipose-heart axis, clinical studies remain largely limited to circulating adipokines [26]. As illustrated in Fig. 6, adipose tissue dysfunction may induce maladaptive adipokine signaling characterized by reduced adiponectin and increased visfatin activity. This imbalance can amplify systemic inflammation, endothelial dysfunction, oxidative stress, and adverse myocardial remodelling, thereby potentially contributing to the development of MACE following AMI.

The observed pattern of lower adiponectin and higher visfatin among patients with MACE is biologically plausible, but the current evidence does not establish that adipokine dysregulation directly contributes to the occurrence of adverse cardiovascular events. Conversely, altered adipokine concentrations may serve as a reflection of heightened baseline disease severity, accentuated systemic inflammation, metabolic dysfunction, endothelial activation, myocardial injury, or post-infarction clinical deterioration. Consequently, reverse causality remains a plausible explanation, particularly because adipokine measurements were obtained at different time points across studies, ranging from the acute AMI phase to later post-infarction periods. The substantial heterogeneity observed for adiponectin further indicates that the pooled estimate may have been influenced by differences in patient characteristics, AMI subtype, comorbidity burden, treatment strategies, follow-up duration, and laboratory measurement protocols.

Consequently, the evidentiary basis of this assertion is deemed to be circumscribed. From a GRADE-oriented perspective, the evidence supporting the association between adiponectin and MACE would likely be rated as low to very low because of the observational study design, small sample size, substantial inconsistency, imprecision, and variability in biomarker assessment. Despite the fact that the visfatin analysis demonstrated a more consistent direction of association and low statistical heterogeneity, the certainty of the evidence remains low. This is due to the fact that the estimate was derived from only two studies and may still be affected by residual confounding and limited generalizability. Consequently, adiponectin and visfatin should currently be regarded as hypothesis-generating biomarkers associated with post-AMI risk, rather than as validated prognostic tools or mechanistic determinants of MACE.

### Reverse causality and post-AMI inflammatory response

An alternative interpretation that should be considered is reverse causality, whereby the observed alterations in circulating adipokines may represent downstream consequences of post-AMI inflammatory and metabolic perturbations rather than upstream determinants of MACE [27, 28]. AMI induces cardiomyocyte necrosis, mitochondrial damage, release of damage-associated molecular patterns, complement activation, and recruitment of neutrophils and monocyte-derived macrophages into the infarcted myocardium [29]. These processes activate systemic inflammatory cascades involving interleukin-1β, interleukin-6, tumor necrosis factor-α, oxidative stress pathways, endothelial activation, and neurohormonal signaling [30, 31]. In this context, altered adiponectin and visfatin concentrations in patients who develop MACE may reflect a more severe inflammatory-metabolic phenotype, greater myocardial injury, impaired infarct resolution, or more advanced cardiometabolic disease rather than a direct causal contribution of adipokine dysregulation to adverse cardiovascular events [32, 33]. Some studies suggest that elevated adiponectin levels in acute coronary syndrome patients may serve as a compensatory mechanism to mitigate ongoing hypoxia-reoxygenation injury and inflammation, potentially confounding prognostic assessments [34].

This phenomenon is further complicated by the reverse epidemiology paradox, where elevated adiponectin in patients with existing cardiovascular disease exhibits an inverse association with survival, possibly due to survival bias or the redistribution of metabolic risk factors following the index event. Moreover, the inherent complexity of adiponectin’s higher-order oligomerization and its role as a ligand complicates simple linear interpretations of these serum concentrations in the post-acute phase [35]. Given the protein’s direct expression in adult cardiomyocytes and its regulatory involvement in autophagic responses to ischemic injury, these variations likely reflect the dynamic state of cardiac remodeling rather than baseline metabolic status [36]. This adaptive upregulation within the infarcted myocardium may serve as a protective response to metabolic inflexibility and impaired fatty acid oxidation [37], though its chronic elevation is paradoxically linked to exacerbated disease severity and mortality in instances of sustained heart failure. Consequently, targeting specific feedback loops, such as the IL6/ADPN/HMGB1 signaling pathway between adipocytes and macrophages within the infarct border zone, remains a critical area for therapeutic modulation to improve clinical outcomes post-MI [38]. Future investigations must clarify whether the divergent systemic levels of adiponectin observed post-infarction represent maladaptive responses or distinct endocrine dysfunction of adipose tissue [39]. Elucidating these mechanisms is essential to determine if adiponectin-targeted therapeutics require precise temporal administration to avoid the detrimental consequences of index event bias [40, 41] Such longitudinal investigations should prioritize characterizing the direct impact of epicardial fat depots on myocardial function, as this tissue frequently exhibits a pathogenic secretory profile in patients with chronic ischemic conditions [42].

### Strengths

This systematic review and meta-analysis has several notable strengths. The study addresses a clinically significant and biologically compelling question by focusing on circulating adipokines as potential markers of adverse cardiovascular outcomes after AMI. In contrast to conventional anthropometric indices such as the body mass index, adipokines have been shown to more accurately reflect the functional and inflammatory state of adipose tissue. This is due to the increasingly recognised role of adipose tissue as a metabolically active organ, involved in vascular inflammation, endothelial dysfunction, and post-infarction remodelling. Synthesising evidence on adiponectin and visfatin, two adipokines with distinct biological profiles, this study provides a more nuanced perspective on the potential relationship between adipose tissue endocrine activity and major adverse cardiovascular events (MACE). The incorporation of BMI as a comparative parameter serves to reinforce the clinical relevance of the analysis, insofar as it enables the findings to be interpreted within the broader context of cardiometabolic risk assessment, which extends beyond the confines of body size alone.

The methodological rigor of this review also serves to strengthen the credibility of the findings. The study was conducted in accordance with PRISMA guidance, registered prospectively in PROSPERO, and based on a systematic search of major biomedical databases. This approach enhances transparency, reproducibility, and reduces the risk of selective reporting. The exclusive inclusion of prospective cohort studies provides a more appropriate temporal framework than cross-sectional evidence for examining the relationship between baseline or early post-AMI adipokine profiles and subsequent clinical outcomes. Furthermore, the methodological quality of the included studies was assessed using the ROBINS-I tool.

### Limitations

Several limitations should be considered when interpreting the present findings. The available evidence remains limited, as only five prospective cohort studies fulfilled the eligibility criteria, with three studies contributing to the adiponectin analysis and two studies to the visfatin analysis. This small evidence base reduced statistical power, widened uncertainty around the pooled estimates, and precluded robust subgroup analyses, sensitivity analyses, or meta-regression to explore potential sources of heterogeneity. Substantial heterogeneity was observed in several analyses, indicating that the pooled effects may have been influenced by differences in patient demographics, ethnicity, AMI subtype, cardiometabolic comorbidities, renal function, inflammatory burden, treatment strategies, follow-up duration, and laboratory measurement protocols. Therefore, the pooled estimates should be interpreted as preliminary signals of association rather than definitive evidence of prognostic utility.

Additional methodological and clinical limitations relate to variability in MACE definitions, adipokine sampling time, residual confounding, and generalizability. Across the included studies, MACE definitions were not fully standardized and variably incorporated cardiovascular death, recurrent myocardial infarction, coronary revascularization, heart failure, stroke, unstable angina, rehospitalization, and all-cause mortality. Such variability may have introduced outcome misclassification and reduced comparability between cohorts, as different MACE components may represent distinct pathophysiological processes and clinical severities. The timing of adipokine measurement also varied from the acute AMI phase to later post-infarction periods, although circulating adipokine concentrations may fluctuate in response to myocardial injury, reperfusion, systemic inflammation, metabolic stress, neurohormonal activation, and recovery. Residual confounding cannot be excluded because adjustment for key variables, including age, sex, diabetes, obesity phenotype, renal function, infarct size, left ventricular function, medication use, and reperfusion strategy, was not uniform across studies. Furthermore, the included cohorts were derived from limited geographic settings, which may restrict the generalizability of the findings to broader AMI populations with different ethnic backgrounds, healthcare systems, cardiometabolic profiles, laboratory standards, and secondary prevention strategies.

### Future directions

Future research should aim to validate these findings in larger, adequately powered, multi-center prospective cohorts using standardized definitions of MACE, harmonized adipokine assays, prespecified blood sampling time points, and comprehensive adjustment for established cardiovascular risk factors and treatment-related variables. Standardization is particularly important because differences in outcome definitions and biomarker measurement protocols may substantially influence effect estimates. Longitudinal studies assessing adiponectin and visfatin trajectories from the acute phase of AMI through post-infarction recovery are needed to clarify temporal relationships and to distinguish whether altered adipokine levels represent potential biological contributors, markers of disease severity, or downstream consequences of systemic inflammation and clinical deterioration.

Further investigations should also evaluate whether adiponectin and visfatin provide incremental prognostic value beyond established clinical risk scores, cardiac biomarkers, imaging parameters, and conventional metabolic indicators. Individual participant data meta-analyses would be valuable to determine whether these associations remain independent after rigorous adjustment and whether they differ across clinically relevant subgroups, including patients with diabetes, obesity, renal dysfunction, STEMI, NSTEMI, or reduced left ventricular ejection fraction. Mechanistic and translational studies are also warranted to clarify the biological pathways linking adipose tissue dysfunction, inflammatory activation, endothelial injury, and adverse post-AMI outcomes. Given the current low certainty of evidence, adipokine profiling should presently be regarded as a hypothesis-generating research direction rather than a clinically validated tool for post-AMI risk stratification.

## Conclusion

This systematic review and meta-analysis showed that lower adiponectin levels and higher visfatin levels were observed among AMI patients who developed MACE. These findings suggest observed associations between circulating adipokine profiles and adverse cardiovascular outcomes following AMI. However, the current evidence is limited by the small number of included observational studies, substantial heterogeneity, and variability in study methodologies. Therefore, the findings should be interpreted cautiously and regarded as exploratory and hypothesis-generating due to very low certainty evidence. 

## Supplementary Information

Supplementary Material 1: Table S1. PRISMA 2020 for Abstracts Checklist.

Supplementary Material 2: Table S2. PRISMA 2020 Checklist.

Supplementary Material 3: Table S3. Search strategy.

Supplementary Material 4: Table S4. Risk of results.

Supplementary Material 5: Table S5. Grade assessment.

Supplementary Material 6: Table S6. Inconsistency and heterogeneity analysis.

## Data Availability

The data that support the findings of this study are available from the corresponding author upon reasonable request.
