# Supplementary material for: Association of adipokines with major adverse cardiovascular events following acute myocardial infarction: a systematic review and meta-analysis
Source: BMC Cardiovasc Disord. 2026 Jun 12;26:725. doi: 10.1186/s12872-026-06120-0 (PMC13488180; doi:10.1186/s12872-026-06120-0)
Supplement: Supplementary file 3 [file 12872_2026_6120_MOESM3_ESM.docx]

**Table S3.** Search Strategy

| **Database** | **Keyword** | **Result** | **Date** |
| --- | --- | --- | --- |
| PubMed | ((((((("adipokines"[MeSH Terms]) OR ("adipokine"[Title/Abstract])) OR ("adipocytokine"[Title/Abstract])) OR ("adiponectin"[MeSH Terms])) OR ("adiponectin"[Title/Abstract])) OR ("visfatin"[Title/Abstract]))  **AND**  (((((((("major adverse cardiovascular event"[Title/Abstract]) OR ("major adverse cardiovascular events"[Title/Abstract])) OR ("adverse cardiovascular event"[Title/Abstract])) OR ("major adverse cardiac event"[Title/Abstract])) OR ("major adverse cardiac events"[Title/Abstract])) OR ("adverse cardiac event"[Title/Abstract])) OR ("adverse cardiac events"[Title/Abstract])) OR ("mace"[Title/Abstract])))  **AND**  ((((("acute myocardial infarction"[Title/Abstract]) OR ("myocardial infarction"[MeSH Terms])) OR ("myocardial infarction"[Title/Abstract])) OR ("percutaneous coronary intervention"[MeSH Terms])) OR ("percutaneous coronary intervention"[Title/Abstract])) | 34 | 25/05/2026 |
| Scopus | TITLE-ABS-KEY ( "adipokine" OR "adipocytokine" OR "adiponectin" OR "visfatin" )  **AND**  TITLE-ABS-KEY ( "major adverse cardiovascular event" OR "major adverse cardiac event" OR "adverse cardiovascular event" OR "adverse cardiac event" OR "MACE" )  **AND**  TITLE-ABS-KEY ( "acute myocardial infarction" OR "myocardial infarction" OR "percutaneous coronary intervention" ) | 44 | 25/05/2026 |
| Cochrane library | "adipokine" OR "adipocytokine" OR "adiponectin" OR "visfatin" in All Text  **AND**  "major adverse cardiovascular event" OR "major adverse cardiac event" OR "adverse cardiovascular event" OR "mace" in All Text  **AND**  "acute myocardial infarction" OR "myocardial infarction" OR "percutaneous coronary intervention" in All Text | 10 | 25/05/2026 |
| Total | | 88 | |
