# Supplementary material for: Association of adipokines with major adverse cardiovascular events following acute myocardial infarction: a systematic review and meta-analysis
Source: BMC Cardiovasc Disord. 2026 Jun 12;26:725. doi: 10.1186/s12872-026-06120-0 (PMC13488180; doi:10.1186/s12872-026-06120-0)
Supplement: Supplementary file 4 [file 12872_2026_6120_MOESM4_ESM.docx]

**Table S4.** Risk of Results

| **Study** | **D1** | **D2** | **D3** | **D4** | **D5** | **D6** | **D7** | **Overall** |
| --- | --- | --- | --- | --- | --- | --- | --- | --- |
| Huang 2010 | Moderate | Serious | Low | Low | Low | Moderate | Moderate | Serious |
| Piestrzeniewicz 2008 | Serious | Moderate | Low | Low | Moderate | Moderate | Serious | Serious |
| Shioji 2007 | Moderate | Moderate | Low | Low | Moderate | Moderate | Moderate | Moderate |
| Hung 2015 | Moderate | Moderate | Low | Low | Moderate | Moderate | Moderate | Moderate |
| Zheng 2020 | Serious | Moderate | Low | Low | Serious | Moderate | Serious | Serious |
