# Supplementary material for: Association of adipokines with major adverse cardiovascular events following acute myocardial infarction: a systematic review and meta-analysis
Source: BMC Cardiovasc Disord. 2026 Jun 12;26:725. doi: 10.1186/s12872-026-06120-0 (PMC13488180; doi:10.1186/s12872-026-06120-0)
Supplement: Supplementary file 5 [file 12872_2026_6120_MOESM5_ESM.docx]

**Table S5.** Grade Assessment

| **Outcome** | **No. of Studies** | **Study Design** | **Risk of Bias** | **Inconsistency** | **Indirectness** | **Imprecision** | **Publication Bias** | **Overall Certainty** |
| --- | --- | --- | --- | --- | --- | --- | --- | --- |
| Adiponectin | 3 | Observational | Serious | Very serious | Not serious | Serious | Undetected | Very Low |
| Visfatin | 2 | Observational | Serious | Not serious | Not serious | Serious | Undetected | Very Low |
| Age | 5 | Observational | Serious | Serious | Not serious | Not serious | Undetected | Low |
| Male Sex | 4 | Observational | Serious | Serious | Not serious | Not serious | Undetected | Low |
| BMI | 5 | Observational | Serious | Very serious | Not serious | Not serious | Undetected | Very Low |
| Diabetes Mellitus | 4 | Observational | Serious | Not serious | Not serious | Not serious | Undetected | Low |
| Hypertension | 4 | Observational | Serious | Not serious | Not serious | Not serious | Undetected | Low |
| Dyslipidemia | 4 | Observational | Serious | Not serious | Not serious | Not serious | Undetected | Low |
| Smoking | 4 | Observational | Serious | Serious | Not serious | Not serious | Undetected | Low |

1. Risk of Bias – All outcomes were derived from observational studies; therefore, a serious risk of bias was assumed due to potential confounding factors and selection bias.
2. Inconsistency – Several pooled analyses demonstrated moderate to high heterogeneity, particularly adiponectin, BMI, age, male sex, and smoking status, resulting in downgrading for inconsistency.
3. Indirectness – The included studies directly evaluated AMI patients with and without MACE and assessed clinically relevant adipokine and cardiometabolic outcomes; therefore, no serious indirectness was identified.
4. Imprecision – Adiponectin and visfatin analyses were downgraded for imprecision because of the limited number of studies and relatively small sample sizes.
5. Publication Bias – Formal assessment of publication bias was limited because fewer than 10 studies were included in each pooled analysis. Therefore, publication bias was considered suspected.

Overall Certainty – Overall certainty of evidence ranged from low to very low, mainly due to observational study design, heterogeneity across studies, and limited sample sizes.
