# Supplementary material for: Association of adipokines with major adverse cardiovascular events following acute myocardial infarction: a systematic review and meta-analysis
Source: BMC Cardiovasc Disord. 2026 Jun 12;26:725. doi: 10.1186/s12872-026-06120-0 (PMC13488180; doi:10.1186/s12872-026-06120-0)
Supplement: Supplementary file 6 [file 12872_2026_6120_MOESM6_ESM.docx]

**Table S6.** Inconsistency and Heterogeneity Analysis

| **Outcome** | **Direction of Effects** | **Heterogeneity (I2, p-value)** | **Variability in Effect Sizes** | **Metodological Considerations** | **Analytical Implications** |
| --- | --- | --- | --- | --- | --- |
| Adiponectin | Lower in MACE compared with non-MACE | I² = 94%,  p < 0.00001 | Effect sizes varied across studies, although most demonstrated lower adiponectin levels in MACE patients | Differences in timing of blood sampling, MACE definitions, and patient characteristics | Findings suggest a possible association with MACE, but interpretation should be cautious due to substantial heterogeneity |
| Age | Higher in MACE compared with non-MACE | I² = 69%,  p = 0.01 | Magnitude of age differences varied between studies | Differences in baseline demographics and inclusion criteria | Older age may contribute to higher cardiovascular risk among MACE patients |
| Male Sex | No significant difference between groups | I² = 62%,  p = 0.05 | Effect estimates varied across studies | Variable sex distribution and limited reporting in some studies | Male sex alone may not consistently differentiate MACE risk |
| BMI | Lower in MACA compared with non-MACE, but not significant | I² = 98%,  p < 0.00001 | Substantial variability in pooled effect sizes | Differences in metabolic status, obesity prevalence, and study populations | BMI alone may inadequately reflect cardiometabolic risk in AMI patients |
| Smoking | No significant difference between groups | I² = 54%,  p = 0.09 | Moderate variability between studies | Definitions of smoking status were not consistently reported across studies | Smoking status may interact with other cardiovascular risk factors rather than independently predict MACE |
